# Structural Characteristics in the γ Chain Variants Associated with Fibrinogen Storage Disease Suggest the Underlying Pathogenic Mechanism

**DOI:** 10.3390/ijms21145139

**Published:** 2020-07-20

**Authors:** Guven Burcu, Emanuele Bellacchio, Elif Sag, Alper Han Cebi, Ismail Saygin, Aysenur Bahadir, Guldal Yilmaz, Marialuisa Corbeddu, Murat Cakir, Francesco Callea

**Affiliations:** 1 Department of Pediatric Gastroenterology Hepatology and Nutrition, Faculty of Medicine, Karadeniz Technical University, Trabzon 61000, Turkey; burcuguven55@gmail.com (G.B.); drturkmen61@gmail.com (E.S.); muratcak@hotmail.com (M.C.); 2 Area di Ricerca Genetica e Malattie Rare, Bambino Gesù Children Hospital, IRCCS, 00165 Rome, Italy; 3 Department of Medical Genetics, Faculty of Medicine, Karadeniz Technical University, Trabzon 61000, Turkey; dralphancebi@yahoo.com; 4 Department of Pathology, Faculty of Medicine, Karadeniz Technical University, Trabzon 61000, Turkey; ismail1974say@hotmail.com; 5 Department of Pediatric Hematology, Faculty of Medicine, Karadeniz Technical University, Trabzon 61000, Turkey; aysenurkbr@yahoo.com; 6 Department of Pathology, Faculty of Medicine, Gazi University, 06170 Ankara, Turkey; drguldal@yahoo.com; 7 Department of Dermatology, Bambino Gesù Children Hospital, IRCCS, 00165 Rome, Italy; marialuisa.corbeddu@opbg.net; 8 Department of Pathology and Molecular Histopathology, Catholic University and Bugando Medical Centre, Mwanza P.O. Box 1464, Tanzania; francesco.callea46@gmail.com

**Keywords:** fibrinogen storage disease, Fibrinogen Trabzon, molecular modelling, folding free energy change, genetics

## Abstract

Particular fibrinogen γ chain mutations occurring in the γ-module induce changes that hamper γ-γ dimerization and provoke intracellular aggregation of the mutant fibrinogen, defective export and plasma deficiency. The hepatic storage predisposes to the development of liver disease. This condition has been termed hereditary hypofibrinogenemia with hepatic storage (HHHS). So far, seven of such mutations in the fibrinogen γ chain have been detected. We are reporting on an additional mutation occurring in a 3.5-year-old Turkish child undergoing a needle liver biopsy because of the concomitance of transaminase elevation of unknown origin and low plasma fibrinogen level. The liver biopsy showed an intra-hepatocytic storage of fibrinogen. The molecular analysis of the three fibrinogen genes revealed a mutation (Fibrinogen Trabzon Thr371Ile) at exon 9 of the γ chain in the child and his father, while the mother and the brother were normal. Fibrinogen Trabzon represents a new fibrinogen γ chain mutation fulfilling the criteria for HHHS. Its occurrence in a Turkish child confirms that HHHS can present in early childhood and provides relevant epidemiological information on the worldwide distribution of the fibrinogen γ chain mutations causing this disease. By analyzing fibrinogen crystal structures and calculating the folding free energy change (ΔΔG) to infer how the variants can affect the conformation and function, we propose a mechanism for the intracellular aggregation of Fibrinogen Trabzon and other γ-module mutations causing HHHS.

## 1. Introduction

Fibrinogen is a secretory glycoprotein synthesized in the liver. The molecule consists of a dimer of heterotrimers, each composed by the polypeptide chains Aα, Bβ, and γ encoded by the genes *FGA, FGB, FGG*. The chains are linked by disulphide bonds and have a central region E connected to two globular regions D (D-E-D) [1].

Fibrinogen plays a major role in coagulation. As a final event in the clot formation, the activation of thrombin results in fibrin monomers that spontaneously aggregate to form fibrils and then the clot [1]. Genetic abnormalities, mostly point mutations, lead to either afibrinogenemia, hypofibrinogenemia or dysfibrinogenemia. The degree of hypofibrinogenemia usually depends upon whether the mutation occurs in a heterozygous, homozygous, or combined heterozygous condition [2]. Mutations can occur with all three genes. A few mutations in the gamma gene (*FGG*) cause abnormal conformation of the molecule, which results into intracellular aggregation and plasma deficiency [3]. This condition, called hereditary hypofibrinogenemia with hepatic storage [4], unlike all other hypofibrinogenemias, is not associated with overt coagulation problems and predisposes invariably to progressive liver disease, similar to α-1-antitrypsin (AAT) deficiency [5].

In this paper, we report on a novel *FGG* mutation, which we examined, in comparison with all previously reported variants causing HHHS. By performing a protein structural analysis and protein folding free energy change (ΔΔG) calculations, which allow us to infer the effects of mutations on the protein structure stability, we discuss the biological implications and the mechanism of intracellular aggregation.

## 2. Case Presentation

### 2.1. Clinical Data

A 3.5-year-old Turkish boy, second born of non-consanguineous parents, was admitted to the Department of Pediatric Gastroenterology and Hepatology Trabzon University because of elevated transaminases. Two weeks earlier, the child had been treated with amoxicillin clavulonate for tonsillitis. The past medical history was unremarkable; in particular, no episodes of bleeding were reported. Upon physical examination, no abnormalities were found. Height and weight were in the normal range. The laboratory test revealed abnormal liver function: ALT = 252 U/L (normal range [*n.r.*] = 15–35 U/L), AST =144 U/L (*n.r.* = 15–35 U/L), γ-GT = 41 U/L (*n.r.* = 7–32 U/L). Protein serum electrophoresis and total bilirubin level were normal. Coagulation test results were aPTT = 35.75 (*n.r.* = 22–35), PT = 19.34 (*n.r.* = 10–15), INR = 1.69 (*n.r.* = 0.8–1.25). Plasma fibrinogen (Clauss method) was very low in two determinations at the one-year interval (36.8 and < 35 mg/dL: *n.r.* = 200–400 mg/dL). Triglycerides were 31.8 mg/dL (*n.r.* = 50–150), cholesterol = 126 mg/dL (*n.r.* = 120–200), HDL = 84 mg/dL (*n.r.* = 45–65). Lipoprotein electrophoresis showed α lipoprotein = 41.9% (*n.r.* = 23–53), β lipoprotein 39% (*n.r.* = 39–70), pre-β lipoprotein = 21.4% (*n.r.* = 5–22), apolipoprotein A1 = 163 mg/mg/dL (*n.r.* = 65–150), apoliprotein A2 = 61 mg/dL (*n.r.* = 25–65). Viral markers and autoantibodies were negative, serum immunoglobulins, α-1-antitrypsin, α-fetoprotein and CPK levels were normal. Liver ultrasound, electrocardiogram, eye examination, tandem mass and urine organic acids tests were normal.

A needle liver biopsy and molecular analysis of the patient were performed with informed consent of the parents. The father showed low fibrinogen plasma levels in two determinations (68.3 mg/dL; 85.5 mg/dL), normal transaminase levels in two determinations (ALT = 17 and 11; AST = 11 and 20) and normal liver structure on ultrasound. Coagulation test revealed mild alterations in the first determination (INR = 1.4, normalized one year later). The child was treated with ursodeoxycholic acid (UDCA) (20 mg/kg/day) for three months without response. Carbamazepine (CZ) was added to the UDCA with decreased transaminases in the first month.

### 2.2. Morphological Studies

The needle liver specimen was fixed in formalin and embedded in paraffin. Four-micron tick sections were stained with hematoxylin and eosin (HE), PAS prior and after diastase (PAS-D) and Masson’s trichrome. Serial sections from the specimens were further stained by immuhistochemistry with commercially available antibodies against fibrinogen, α-1-antitrypsin, and albumin as previously described [6].

**Figure 1:**
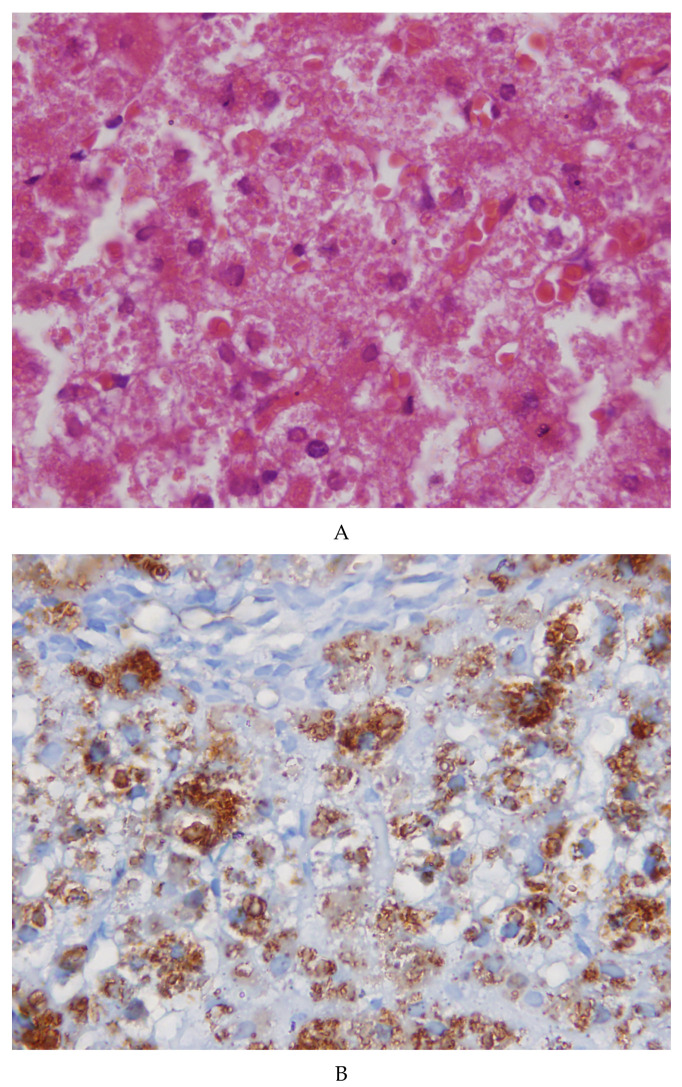
(**A**) Sections from the patient’s liver biopsy. The microphotograph shows a normal lobular architecture with no inflammation or necrosis. Hepatocytes contain round eosinophilic inclusions at times surrounded by a clear halo (×40). (**B**) The inclusions show a strong positivity for fibrinogen on immunostaining (×20).

The needle liver biopsy showed preserved lobular architecture. Hepatocytes contained cytoplasmic inclusions appearing as round or polygonal eosinophilic bodies, sometimes surrounded by a clear halo (Figure 1A). The inclusions were negative on PAS and PAS diastase (PAS-D) staining. The inclusions were positive on immunostaining for fibrinogen (Figure 1B) and negative for α-1-antitrypsin and albumin. The positivity for fibrinogen was selective and exclusive. Masson’s trichrome revealed a very mild portal fibrosis. No signs of inflammation or necrosis.

### 2.3. Molecular Analysis

DNA sequencing of the three fibrinogen genes was carried out on DNA extracted from peripheral blood from all four family members as previously described [6]. This analysis revealed in the proband and father a mutation at exon 9 of the FGG, corresponding to the Thr371Ile change in the protein. Molecular analysis in the proband’s mother and sister was normal.

### 2.4. Protein Structural Analysis

**Figure 2:**
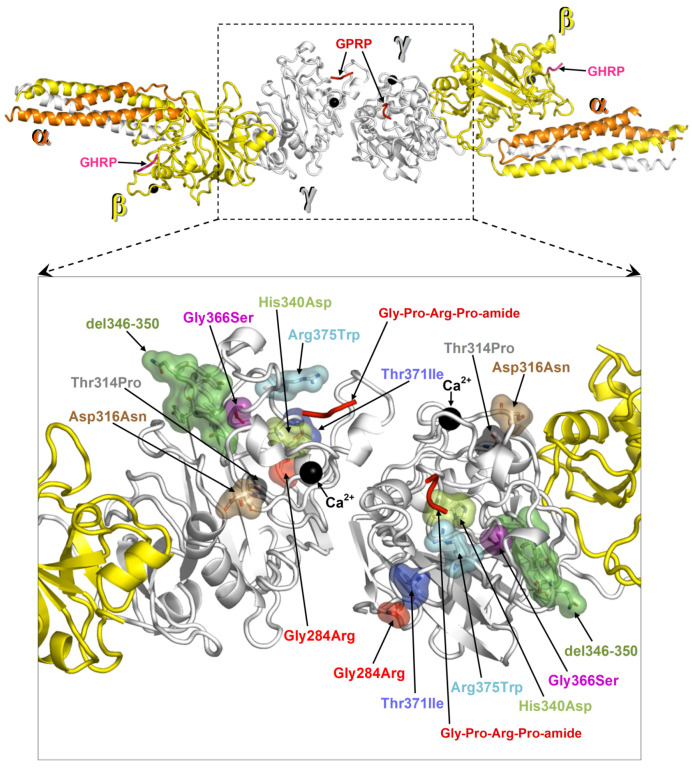
Map of fibrinogen γ chain mutations on the structure of fragment double-D. Structure of fragment double-D from human fibrin Protein Data Bank (PDB 1FZC) with the amino acid residues affected by mutations known to cause fibrinogen hepatic storage. The bound calcium ions are shown as black spheres, the peptide Gly-Pro-Arg-Pro-amide (GPRP, an analogue of the α chain “knob”) bound to the “hole” of γ chains is represented in red ribbons, and the peptide Gly-His-Arg-Pro-amide (GHRP, an analogue of β chain “knob”) bound to the “hole” of β chains is in viola ribbons.

Structural analysis was performed on the crystal structure of fragment double-D from human fibrin (Protein Data Bank, PDB, entry 1FZC). ΔΔG values were calculated with FoldX [7] for one amino acid replacement at a time and for each of the two fibrinogen γ chains represented in the structure of fragment double-D. ΔΔGs were averaged over 5 runs on each mutation. Prior to ΔΔG calculations, the PDB repair utility was applied to the crystal structure. The mutation Thr371Ile localizes in the globular domain of the γ-module near the hole that spontaneously receives the new N-terminal (Gly-Pro-Arg peptide) of the proteolyzed fibrinogen α chain. The localization of all γ chain mutations so far identified is shown in Figure 2. The protein folding free energy changes (ΔΔG) calculated for each mutation is reported in Table 1. The mutations Gly284Arg and Gly366Ser presented the highest ΔΔGs values, Asp316Asn and the mutation from the proband Thr371Ile presented the two lightest ΔΔGs values. Asp316Asn was located near a calcium binding site (Figure 2), whilst Thr371Ile (ΔΔG of 0.7 and 0.8 Kcal/mol individually mutating one or the other γ chain in fragment double-D) was located in proximity of the “hole”. The remaining mutations with higher ΔΔG values (Table 1) were located in regions critical for fibrinogen structure (Figure 2).

The protein folding free energy change (ΔΔG) calculated for each missense mutation is reported in Table 1.

## 3. Discussion

In this work, we present novel clinical–pathological findings on the fibrinogen γ chain Thr371Ile variant reported by Brennan et al. [8] who already highlighted the structural role of the mutation in destabilizing the γ-module thus potentially promoting either intracellular degradation or intracellular inclusion formation [8]. The authors have proposed the name Muncien for that fibrinogen. In our Turkish child patient carrying the same mutation, we have found within the hepatocytes the characteristic eosinophilic inclusions that have turned out to be exclusively and selectively immunoreactive for fibrinogen, similar to all other γ chain mutations so far published: Brescia [3,4], Aguadilla [9], Angers [10], Al du Pont [11], Pisa and Beograd [12], Ankara [13]. Thus, the mutation Thr371Ile in the γ chain represents a new variant of fibrinogen resulting in HHHS. According to the custom, we have proposed the designation Fibrinogen Trabzon for the new Turkish variant. Fibrinogen Trabzon represents the third Turkish variant. Turkey appears to be the country with more mutations (Aguadilla, Ankara and Trabzon), while Aguadilla seems to be the most frequent mutation worldwide. Because of the increasing number of fibrinogen γ chain mutations resulting in hepatocellular storage, we have performed the structural analysis of Fibrinogen Trabzon and we have demonstrated the mechanism of the intracellular aggregation by comparing its conformational abnormality with all other mutations in the γ-module, and by calculating the protein folding ΔΔG values associated with each amino acid variant. The mutation Thr371Ile is localized near the “hole” of the fibrinogen γ chain that spontaneously receives the new N-terminus (Gly-Pro-Arg peptide) of the fibrinogen α chain, representing the “knob”, that is generated upon proteolysis by thrombin during clotting. The importance of this region has also been demonstrated by synthetic peptides capable of binding the “hole”, which compete with the true “knob” of fibrinogen α chain and inhibit polymerization [14]. Thus, the Thr371Ile variant might have effects on the “hole” conformation. We propose that an inhibitory effect on polymerization can also arise upon alterations of the “hole” conformation in the Thr371Ile variant. However, the damage caused by this variant might occur at an earlier stage than polymerization since Sheen et al. showed the failure of export of the fibrinogen γ-module carrying Thr371Ile or other variants in the *Pirchia Pastoris* cell supernatant [15].

Our clinical data provide evidence that the Thr371Ile amino acid change belongs to the so far restricted group of mutations causing hepatic storage. Based on our structural analysis, we rationalize a common mechanism for Thr371Ile and other variants of fibrinogen γ chain known to give rise to the hepatic storage phenomenon, Brescia Gly284Arg [3,4], Aguadilla Arg375Trp [9], Angers G346_Q350del [10], Al Du Pont Thr314Pro [11], Pisa Asp316Asn and Beograd Gly366Ser [12], and Ankara His340Asp [13]. As we proposed in a previous study, particular mutations cause a failure in the fibrinogen assembly due to conformational changes in the γ-module and consequent abnormal over-exposure of protein hydrophobic patches that could act as seeds for the aggregation of hydrophobic molecules [16]. All mutations cluster near the interface of dimerization (Figure 2). We calculated the protein folding free energy change (ΔΔG) between each mutant and the wild type (Table 1). Two mutations, Gly284Arg and Gly366Ser, present very high ΔΔGs, indicating heavy effects on the stability of the domain. Apparently, the Asp316Asn change, which presents the less significant ΔΔG value, might seem to have little or no effect on the fibrinogen γ chain structure. However, Asp316Asn is near a calcium-binding site where even small structural alterations may affect the ability to bind this cofactor, hence producing more deleterious conformational effects than can be foreseen by the ΔΔG result. The second lightest ΔΔG value is presented by the Thr371Ile variant (ΔΔG of 0.7 and 0.8 Kcal/mol individually mutating one or the other γ chain in fragment double-D). However, its proximity to the “hole”, despite the predicted moderate destabilization, can become critical as it alters this ligand binding region. The same holds true for the remaining mutations, His340Asp and Arg375Trp that are located nearby the “hole”. Therefore, for all hepatic storage-associated variants, disruptive effects on the protein can be envisaged either by the significant structural changes predicted by ΔΔG values (Gly284Arg and Gly366Ser) and/or because the affected amino acids clearly fall in regions critical for the fibrinogen function (Asp316Asn, Thr371Ile, His340Asp, Gly366Ser, and Arg375Trp).

Intriguingly, all mutations locate near, but not inside, the interface of homodimerization of two fibrinogen γ molecules. As we showed previously, this interface and other protein regions contain hydrophobic patches that could remain abnormally exposed upon mutation-caused failure in the dimerization of two Aα-Bβ-γ fibrinogen heterotrimers, therefore the mutated γ chain might work as a seed for phenomena of aggregation of hydrophobic peptides and lipid [16]. Previous studies have shown that the mutant γ chains are entirely retained within the hepatocytes [17] and that they are not found in circulation either in Brescia [3], Aguadilla [9], Al du Pont [11], or Thr317Ile (Muncien) [8]. This study has shown that the mutation Thr371Ile implies a very mild effect on the stability of the globular γ-module. We wonder whether this observation could serve to set up a suitable experimental model aimed to inhibit aggregation or enhance degradation by using small molecules in analogy with Z AAT [18]. Presently, the attempt to reduce the storage by activating the autophagy/proteasome systems has given controversial/unsatisfactory results [19,20,21].

The detection of the new mutation in the fibrinogen γ chain, namely Fibrinogen Trabzon, is elongating the list of mutations resulting in hepatic storage. Fibrinogen γ chain mutations with hepatic storage have been detected in Europe, U.S., the Caribbean, Saudi Arabia, the Far East and Turkey. Interestingly, all mutations have been described in the heterozygous condition, thus suggesting an autosomal dominant modality of transmission, either haploinsufficiency or negative dominant, and indirectly indicating the incompatibility of the life of the homozygous state—hence, explaining why the disease is exceedingly rare.

All the mutations have been identified following the histological finding of accumulation in liver tissue sections, mostly in children presenting with liver test dysfunction and histopathological alterations ranging from mild changes to cirrhosis [3] even in early childhood [6,22,23,24]. This case offers the opportunity to discuss two relevant points neglected in all previous reports on fibrinogen storage diseases: a) the concomitance of normal transaminase levels and hypofibrinogenemia due to gamma chain mutations, b) the correlation genotype/phenotype. Indeed, this condition has been observed in a variable number of family members with Brescia [3,24], Aguadilla [9,20,22,25], Ankara [13], Angers [10], Pisa and Beograd [12] mutations. The phenomenon is analogous to what happens in individuals with AAT deficiency who may not develop any clinical sign of liver disease, despite the fact that all of them invariably accumulate the protein [26] and that the storage causes a toxic cell damage [27]. The unavoidable storage process represents the elementary lesion and consequently the true phenotype that strongly correlate with the mutations in both AAT deficiency and FSD [27]. A further interesting point is the absence of overt coagulation disturbances in most affected patients, despite the constantly low fibrinogen levels. While the liver pathology is well explained by the accumulation of fibrinogen in the liver cells of patients with *FGG* mutations, the mild or null clinical impact towards the coagulation process in the vast majority of these patients remains to be clarified. The discovery of the hepatic storage secondary to *FGG* mutations has allowed us to classify the entity as an ERSD and to attribute an etiology to a number of liver cirrhosis that otherwise would remain cryptogenic.

## 4. Conclusions

The mutation Thr371Ile (Fibrinogen Trabzon) is elongating the list of fibrinogen γ chain mutations capable of provoking hepatic storage and hypofibrinogenemia. For this mutation as well as for all other hepatic storage associated variants, the disruptive effect on the protein structure can be envisaged either by the destabilization predicted by the free energy changes in protein folding and/or by because the affected amino acids fall in regions critical for fibrinogen structure. The study definitely indicates that in Fibrinogen Trabzon as well as in all other mutations causing HHHS, there is a strong genotype/phenotype correlation as the true phenotype consists in the unavoidable hepatic storage that represents the elementary lesion and the specific marker of the disease.

## Figures and Tables

**Table 1:** Free energy change (ΔΔG) in protein folding of fibrinogen γ amino acid variants.

Variant	ΔΔG (kcal/mol)	
	γ Chain C*	γ Chain F*
Thr371Ile	0.7 (0.1)	0.8 (0.3)
Gly284Arg	10.5 (0.4)	14.4 (0.9)
Arg375Trp	1.9 (1.2)	2.1 (1.0)
Thr314Pro	1.0 (0.3)	2.5 (0.0)
Asp316Asn	−0.3 (0.1)	0.1 (0.1)
Gly366Ser	6.3 (0.8)	4.4 (0.2)
His340Asp	2.8 (0.3)	2.2 (0.0)

The ΔΔG values of fibrinogen γ mutations calculated on the crystal structure of fragment double-D from human fibrin (PDB structure 1FZC). The ΔΔGs are the average of five FoldX calculations on each amino acid replacement on each of the two fibrinogen γ chains (standard deviations in parenthesis). Positive and negative ΔΔG values, respectively, imply destabilizing and stabilizing effects of the mutations on the γ chain structure. C* and F* are the names of the two γ chains as in the records of the above PDB entry.
